# Implications of *Porphyromonas gingivalis* peptidyl arginine deiminase and gingipain R in human health and diseases

**DOI:** 10.3389/fcimb.2022.987683

**Published:** 2022-09-29

**Authors:** Yoke Chan Chow, Hok Chai Yam, Baskaran Gunasekaran, Weng Yeen Lai, Weng Yue Wo, Tarun Agarwal, Yien Yien Ong, Siew Lee Cheong, Sheri-Ann Tan

**Affiliations:** ^1^ Department of Bioscience, Faculty of Applied Sciences, Tunku Abdul Rahman University College, Kuala Lumpur, Malaysia; ^2^ Department of Biotechnology, Faculty of Applied Sciences, UCSI University, Kuala Lumpur, Malaysia; ^3^ Department of Pharmaceutical Chemistry, School of Pharmacy, International Medical University, Kuala Lumpur, Malaysia; ^4^ Department of Biotechnology, Koneru Lakshmaiah Education Foundation, Guntur, India

**Keywords:** *Porphyromonas gingivalis*, peptidyl arginine deiminase, gingipain R, citrullination, systemic disease, inhibitors

## Abstract

*Porphyromonas gingivalis* is a major pathogenic bacterium involved in the pathogenesis of periodontitis. Citrullination has been reported as the underlying mechanism of the pathogenesis, which relies on the interplay between two virulence factors of the bacterium, namely gingipain R and the bacterial peptidyl arginine deiminase. Gingipain R cleaves host proteins to expose the C-terminal arginines for peptidyl arginine deiminase to citrullinate and generate citrullinated proteins. Apart from carrying out citrullination in the periodontium, the bacterium is found capable of citrullinating proteins present in the host synovial tissues, atherosclerotic plaques and neurons. Studies have suggested that both virulence factors are the key factors that trigger distal effects mediated by citrullination, leading to the development of some non-communicable diseases, such as rheumatoid arthritis, atherosclerosis, and Alzheimer’s disease. Thus, inhibition of these virulence factors not only can mitigate periodontitis, but also can provide new therapeutic solutions for systematic diseases involving bacterial citrullination. Herein, we described both these proteins in terms of their unique structural conformations and biological relevance to different human diseases. Moreover, investigations of inhibitory actions on the enzymes are also enumerated. New approaches for identifying inhibitors for peptidyl arginine deiminase through drug repurposing and virtual screening are also discussed.

## Introduction

**Figure 1:**
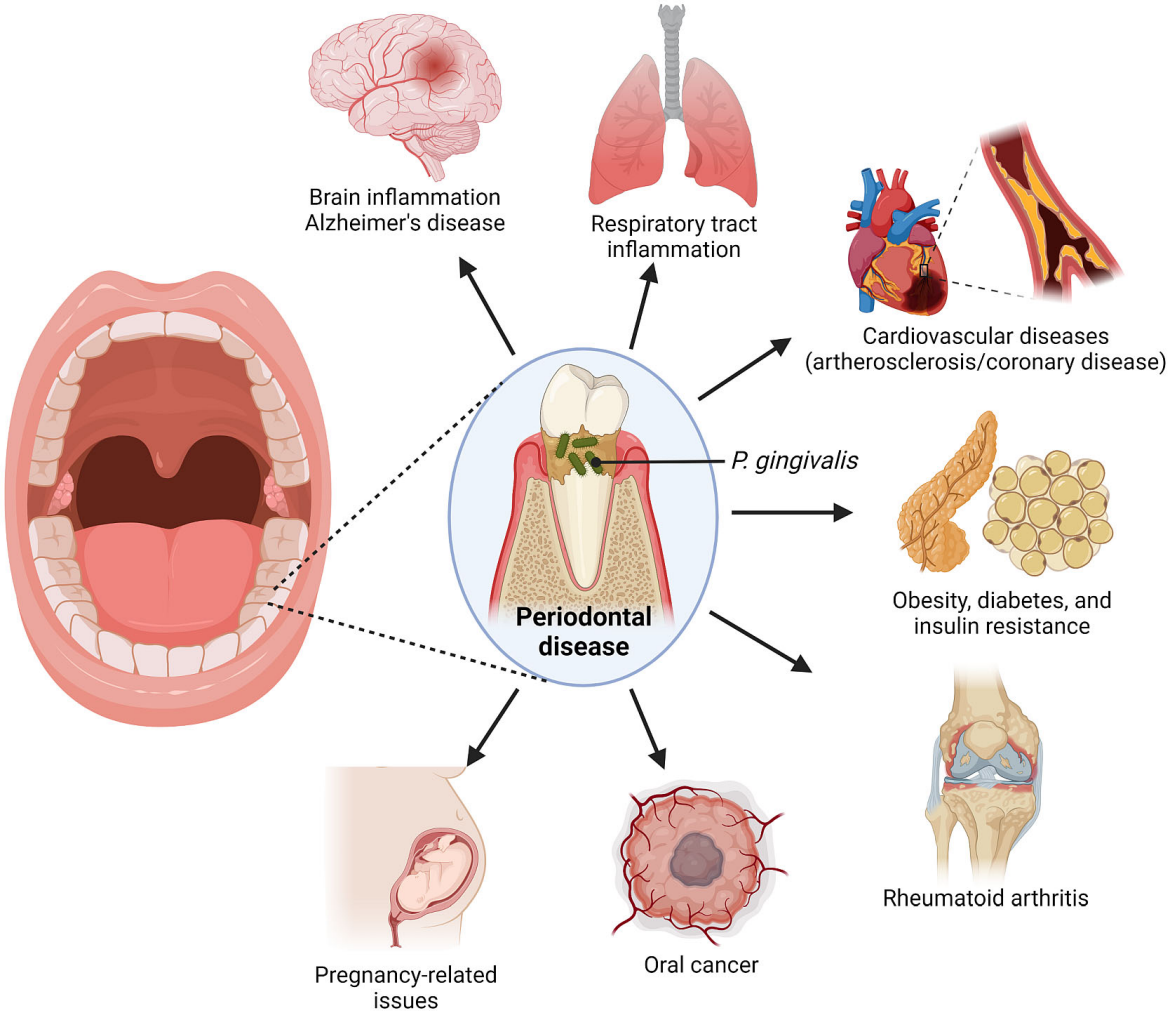
Link between *P. gingivalis*, an oral pathogen, with various systemic diseases.

*Porphyromonas gingivalis* is a gram-negative, anaerobic coccobacillus that is commonly present in the oral cavity of patients with poor oral health (Bostanci and Belibasakis, 2012). It is majority associated with pathogenesis of periodontal diseases, such as periodontitis (PD), which is a highly prevalent inflammatory infection of the gums and gingival tissue characterized by progressive destruction of tissues supporting the tooth (Howard et al., 2021; Zhang et al., 2021). While the disease is exacerbated by a large number of pathogens that reside in the gingiva, studies have been able to point out *P. gingivalis* as a major pathogen in such oral disease (Bostanci and Belibasakis, 2012; Olsen and Yilmaz, 2019; Hu et al., 2020). Production of *P. gingivalis* biofilm has been linked to the formation of bacterial plaques in the gingiva tissue, which further promotes gum injury by other oral bacteria. (Fiorillo et al., 2019). Recent studies have also associated *P. gingivalis* with various other systemic diseases, including Alzheimer’s, orodigestive cancers, and rheumatoid arthritis (Olsen et al., 2018) (**Figure 1**).

In fact, the prevalence of PD in rheumatoid arthritis (RA) patients has been broadly documented. When compared to cohorts without rheumatic inflammations, RA patients have been found to have severe PD. An epidemiological study by Rodríguez-Lozano et al. reported that 44.92% of 187 RA patients were afflicted with PD, with early RA (ERA) patients having a considerably higher rate of periodontal disease (31.43%) than those with established RA (9.21%) (Rodríguez-Lozano et al., 2019). Besides, another recent research had linked PD to the Alzheimer’s disease (AD), remarking that AD patients possessed significantly more plaques and fewer number of teeth as compared to the control group with no AD (de Oliveira Araújo et al., 2020). In accordance with keystone-pathogen hypothesis, *P. gingivalis* is considered the main pathogen that instigated the progression of these diseases (De Vries et al., 2021).

Conventional treatment for PD includes both non-surgical as well as surgical interventions based on the severity of the disease. For mild to moderate cases, non-surgical treatment such as mechanical periodontal therapy by scaling and root planing is usually recommended. Nevertheless, these gold standard therapies do have limitations. It has been reported that SRP may cause translocation of periopathogens from one tooth pocket to another which leads to reinfection after treatment (Apatzidou and Kinane, 2009). Furthermore, this technique will not remove bacterial infiltration on other periodontal tissues besides the periodontal pocket (Lu et al., 2022). Hence, antibiotics supplementation (eg: metronidazole and amoxicillin) is sometimes prescribed along with this treatment option to enhance its efficacy but its usage is limited due to antibiotic resistance among the periopathogens (Lu et al., 2022; Pretzl et al., 2018). Therefore, novel strategies should be implemented since there are many reports linking PD with other diseases.

In recent years, the mechanism employed by *P. gingivalis* to cause multiple types of infections while evading the host immune system has actively been investigated. This bacterium possesses a plethora of virulence factors that enable it to evade the host’s immune defenses (**Table 1**). Virulence factors such as gingipain R (RgpA and RgpB) and a peptidylarginine deiminase (PPAD) could further cause dysregulation to the host immune system by increasing the inflammatory responses (Konig et. al., 2014; Karkowska-Kuleta et al., 2020). Gingipains contribute to large portion (85%) of the proteolytic actions and express 99% of “trypsin-like” activities of *P. gingivalis*. These activities allow *P. gingivalis* to degrade host proteins for nutrients as well as those proteins which are responsible for immune activities (e.g. plasma proteins, extracellular matrix proteins, cytokines and host cell surface proteins) (Li and Collyer, 2011). Besides, production of outer membrane vesicles (OMV) that serve to transport molecular effector related to host immune dysregulation is also associated with gingipains (Zhang et al., 2016; Okamura et al., 2021). Inhibition of the gingipains is shown to reduce hemolytic activities and biofilm formation of *P. gingivalis*, thus limiting its virulency (Tantivitayakul et al., 2020).

**Table 1:** Virulence factors present in *P. gingivalis*.

Virulence factors	Actions	References
Gingipains	* Enhance vascular permeability * Evade host immune systems by immunoglobulin degradation * Degrades periodontal tissues * Cleaves immune cell receptors	Li and Collyer (2011); Nakayama and Ohara (2017)
Peptidylarginine deiminase	* Citrullination of host and endogenous proteins * Generation of ammonia to protect *P. gingivalis* from acidic environments	Goulas et al. (2015); Bereta et al. (2019)
Lipopolysaccharide	* Cause host inflammation through induction of macrophages to release the proinflammatory mediators * Exhibits bone resorption activity	Kumada et al. (1995); Zhang et al. (2018)
Fimbriae (FimA, Mfa1)	* Biofilm formation * Allows interaction between *P. gingivalis* and other oral bacteria	Lee et al. (2018); Kan et al. (2019)
Hemagglutinins	* Promotes adherence to host tissues * Promotes platelet aggregation * Lysis of erythrocytes	Eltigani et al. (2019); Kan et al. (2019)
Type IX secretion system (T9SS)	* Expresses gingipains on cell surfaces * Secretes iron chelating proteins * Transports virulence factors to cell surface	de Diego et al. (2016); Benedyk et al. (2019),
Capsules	* Evades phagocytosis and invasion by keratinocytes * Facilitates in biofilm formation	de Diego et al. (2016); Benedyk et al. (2019),

To date, both gingipains and PPAD have garnered significant interest among researchers due to their possible correlations and synergistic effects in causing systemic diseases to the host organisms. Particularly, PPAD causes citrullination of the host proteins, mainly fibrinogen and α‐enolase, by converting arginine residues to citrullinated residues which are essential for many physiological processes including OMV biogenesis in addition to triggering other pathological inflammatory conditions, leading to breakdown of immune tolerance to diseases other than PD (Wegner et al., 2010; Maresz et al., 2013; Vermilyea et al., 2021). On the other hand, Rgp facilitates this process by cleaving the fibrinogen and α‐enolase to expose the C-terminal arginine for PPAD to citrullinate. This leads to creation of neoepitopes and generation of autoantibodies against the citrullinated peptides, giving rise to abnormal autoimmune response (Gully et al., 2014) as illustrated in **Figure 2**. Occurrence of aberrant citrullination caused by the PPAD can be inhibited and thus, the development of PPAD-associated systemic diseases could be prevented to an extent.

**Figure 2:**
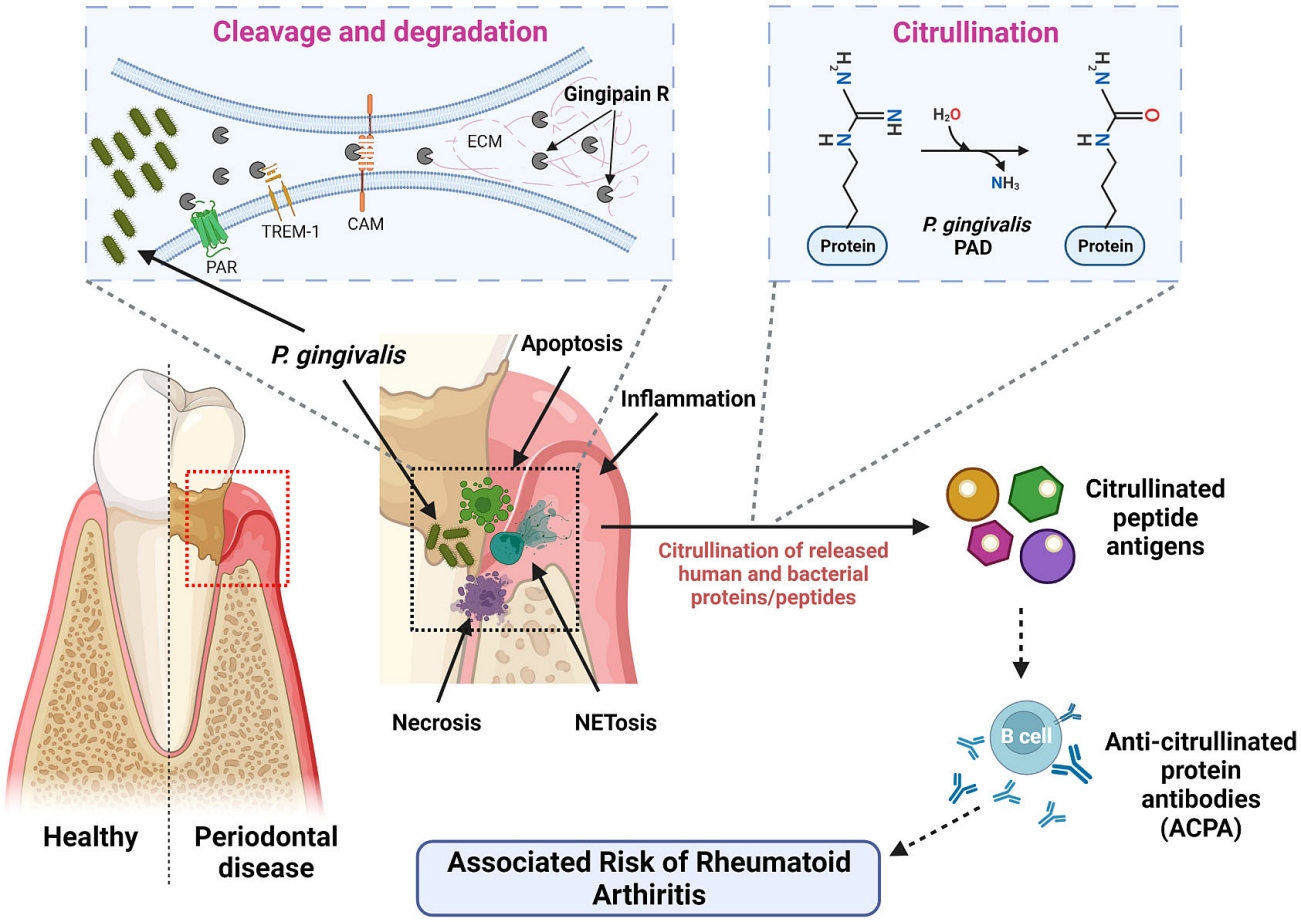
Mechanism of action of *P. gingivalis* virulence factors – Rgp and PPAD. *P. gingivalis* invades by secreting gingipains, which shred and degrade different cell receptors and ECM components. This disrupts signaling pathways and promotes tissue damage. PPAD and human PAD then citrullinate the liberated peptides. These citrullinated peptide antigens are recognized by antigen-presenting cells, resulting in the generation of anti-citrullinated protein antibodies (ACPAs) by the B cells. These ACPAs are associated with an increased risk of RA.

Given the potential of both virulence factors as therapeutic targets of human diseases, this review has focused on the structural properties of Rgp and PPAD as well as their therapeutic relevance. In addition, up-to-date findings on inhibition of the virulence factors by natural products and synthetic compounds are elaborated in the later sections to provide a comprehensive overview on how *P. gingivalis*-related disorders may be overcome. Furthermore, recommendation of new approaches for identifying inhibitors for the virulence factors, especially PPAD are also discussed. These inhibitors could be used as adjuncts to the conventional therapies.

## Gingipain R

### Structural biology of Rgp

Rgps are the major virulence factors of the *Porphyromonas gingivalis*. They are cysteine proteinases that are expressed by two related genes, rgpA and rgpB (Pavloff et al., 1995). The rgpA-encoded gingipains occur as single-chain RgpA proteinase or as high-molecular-mass HRgpA, a 95 kDa non-covalent complex consisting of a 50 kDa catalytic domain with a haemagglutinin (adhesion) domain (Rangarajan et al., 1997). The rgpB gene, on the other hand, codes for a 507-residue protein and lacks the haemagglutinin domain; its mature C-terminally shortened product, RgpB, a 435-residue single-chain protein that is identical to RgpA, shows considerable divergence from position 363 onwards (Mikolajczyk-Pawlinska et al., 1998; Potempa et al., 1998). Such mature and soluble protein is organized into a catalytic domain (residues 1-351) and an immunoglobulin-like domain (residues 352-435) (Eichinger et al., 1999). Both Rgps A and B show specificity towards Arg-Xaa peptide bonds, of which their hydrolytic activity is activated by reducing agent, such as cysteine.

**Figure 3:**
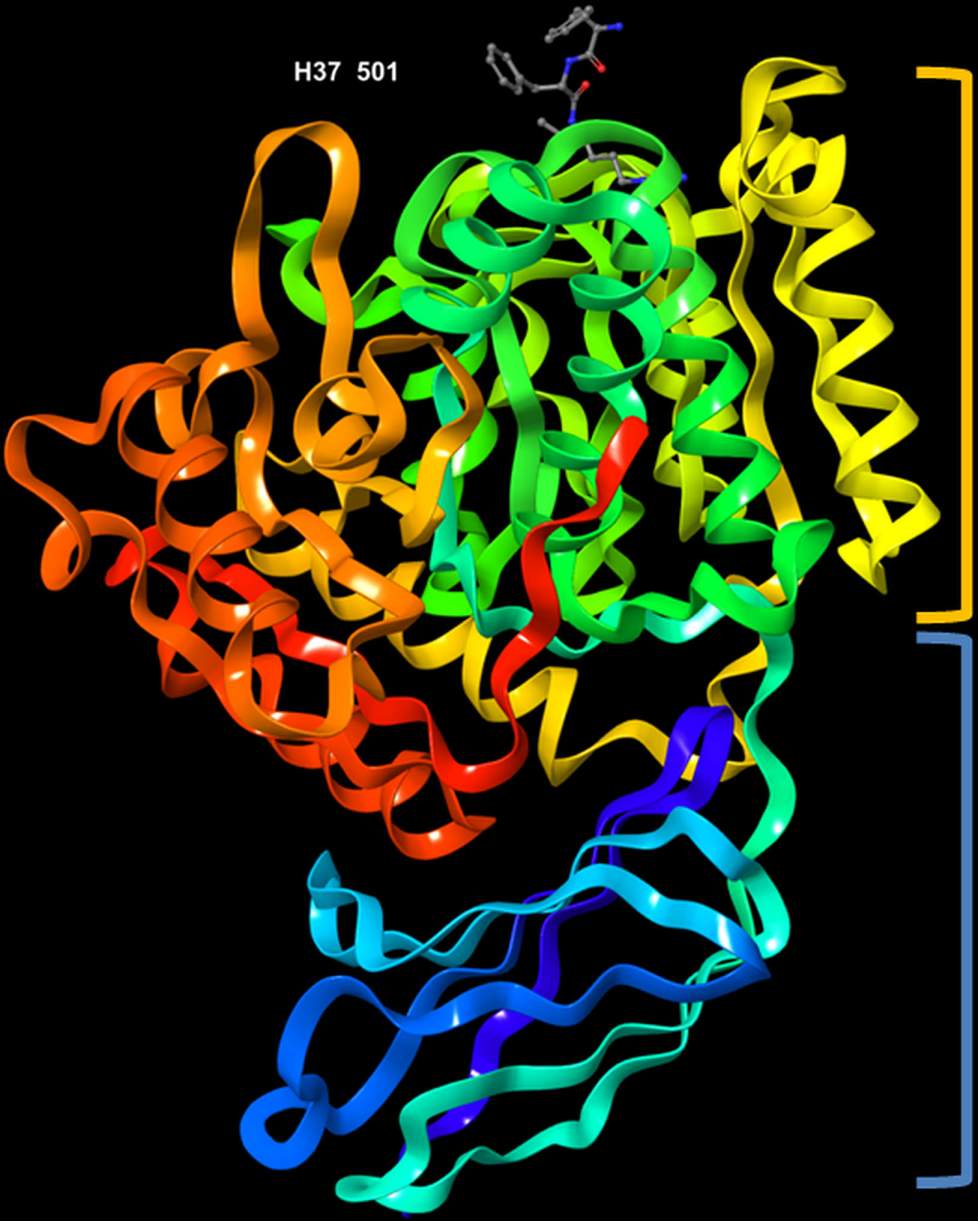
3D representation of *Porphyromonas gingivalis* Rgp with co-crystallized ligand, D-Phe-Phe-Arg-chloromethylketone, labelled H37 501 (PDB ID: 1CVR). Highlighted in orange: crown (catalytic domain), blue: root (IgSF domain).

The crystal structure of RgpB (PDB entry: 1CVR), with a resolution of 1.5 Å, was first determined by Eichinger et al. (1999). The protein is made up of an almost spherical ‘crown’ and a ‘root’. The crown represents the catalytic domain, which consists of the N-terminal 351 residues, whereas the root comprises an immunoglobulin superfamily (IgSF) domain (**Figure 3**). It has high proportion of regular secondary structures; the catalytic domain possesses structural motif of a typical α/β protein, while the IgSF domain acquires an all-β conformation. The catalytic domain consists of two sub-domains, namely sub-domain A and B. Each of them comprises strands of β-sheets sandwiched by α-helices, which is the characteristic for α/β open-sheets structure. Sub-domain A has four parallel strands twisted regularly. The core of the domain is mainly hydrophobic in nature. It is linked to sub-domain B through a short surface-located segment. The sub-domain B is made up of six strands that are flanked by seven helices h5-h11. The last innermost strand (s10) subsequently enters the IgSF domain, which resembles an elongated seven-stranded β-barrel (Eichinger et al., 1999). Its function is not well documented thus far although there is increasing number of evidence that indicate its role in stabilizing the structure of the catalytic subdomain (Potempa et al., 2003).

The active site of RgpB is relatively flat, and characterized by a negative electrostatic potential. It is surrounded by strand His211-Glu214, the rising segment Ala243-Val245, the s9-h9 connecting segment and the perpendicularly twisted s5-h5 (from Glu152 to Asp163) in the sub-domain B. Among the residues, Cys244 is located in the centre of the active site. Of note, the co-crystallized peptide inhibitor, D-Phe-Phe-Arg-chloromethylketone (FFRCMK) was found bound to the Cys244. A deep S1 pocket is present in close proximity to the Cys244, of which the Val242 and Thr209 Cγ atoms form the base, while the summit of the pocket is enveloped by the indole moiety of Trp284. At the bottom of the pocket, Asp163 carboxylate group acts as a charge anchor. In addition, the upper and lower side of the pocket is also lined by the side chain of Met288 and by segment Gly210-His211 and His211 side chain, respectively (Eichinger et al., 1999). Specifically, the Cys244-His211 catalytic dyad acts to bind and cleave the Arg-Xaa substrate. The side chain of residue Asp163 is observed to form a salt bridge with the guanidyl group of Arg in the FFRCMK inhibitor. Some hydrogen bonds were also found connecting the inhibitor to residues Gly212, Gln282 and Trp284 that are also close to the active site (Eichinger et al., 1999; Li and Collyer, 2011). Furthermore, a calcium ion placed just below the S1 pocket was found to clamp the helices h5 and h9 together; it is believed that the removal of this calcium ion may result in shift or disordering of Asp163 and can ultimately affect the hydrolytic activity (Eichinger et al., 1999).

In general, the S1 pocket is a deep and narrow slot with a hydrophobic lid; it is mainly delineated by hydrogen bond acceptors that largely explains the preference of RgpB for P1-Arg residues. Such negatively charged S1 pocket and active site further corroborates the specificity of RgpB for P1-Arg-containing peptides (Potempa et al., 1997; Grenier et al., 2003; Barrett et al., 2013). Particularly, Met288 in the active site is considered as a key factor in discriminating substrate specificity between Rgp and Kgp (gingipain K, a cysteine proteinase from the same bacterium *Porphyromonas* gingivalis that shows specificity for Lys-Xaa peptide bonds). Based on the sequence alignment reported by Pavloff et al. (1997), such Met288 is replaced by a tyrosine residue in Kgp, of which the side chain phenol group might be rotated into the S1 pocket and form a hydrogen bonding with the ammonium group of an inserted P1-Lys. On the other hand, the Met288 Sδ in Rgp was found to engage in a hydrogen bonding with the adjacent guanidinium group of an inserted P1-Arg through one of its lone pair orbitals.

### Association of Rgp with non-communicable and infectious diseases

Rgp plays a critical role in many non-communicable diseases (**Table 2**) that are currently on the rise; these include the diseases of oral cavity, such as PD (Wolf et al., 2021). The Rgp triggers gingival crevicular fluid exudation, edema formation, leukocyte accumulation, and gingival inflammation (Imamura, 2003). These symptoms are facilitated by the conversion of prothrombin to thrombin by Rgp, in which leukocyte chemotaxis is instigated (Hosotaki et al., 1999; Imamura et al., 2001). Furthermore, Rgp degrades proinflammatory cytokines (like IL-1β, TNF-α, IFN-γ, IL-12, IL-8, IL-6) and IL-6 receptor, impairing inflammation-associated host defense mechanisms. It also activates matrix metalloproteinases (MMPs) such as MMP-1, MMP-9, and MMP-3, which degrades collagens fibers attached to the root surface and damages periodontal ligament (Fitzpatrick et al., 2009). Taken together, through the activation of kallikrein/kinin pathway, dysregulation of clotting and fibrinolytic pathways, and disruption of the host defense system, Rgp results into the development of PD. In atherosclerosis condition, Rgp is associated with plague formation in the arteries, which is made up of plasma lipoproteins, particularly low-density lipoproteins (LDL) (Spiller, 1996). Rgp degrades two apoliproteins, apoE and apoB-100, interfering with the interaction of apoE and LDL receptors on hepatocytes, whereas apoB-100 degradation leads to an increased LDL uptake by macrophages, which can result in the formation of atherosclerotic plaque (Hashimoto et al., 2006; Lönn et al., 2018). This is aided further by Rgp-induced reactive oxygen species and LDL peroxidation, which contributes to the prevalence of atherosclerosis. (Lönn et al., 2018). Dysregulation of Angpt1 and Angpt2 expressions by Rgp are also linked to progression of atherosclerosis (Post et al., 2008; David et al., 2009; Zhang et al., 2015). Angpt1 and Angpt2 regulate inflammatory responses in different ways, with Angpt1 serving as an anti-inflammatory regulator and Angpt2 acting as a proinflammatory regulator. Rgp increases the expression of Angpt2 while decreasing the expression of Angpt1 in human aortic smooth muscle cells, inducing their migration (Zhang et al., 2015), a crucial event in the pathophysiology of atherosclerosis (Rudijanto, 2007). As a whole, Rgp is implicated in cardiovascular disorders by modifying vascular LDL/VLDL and HDL into atherogenic forms and regulating the production of Angpt1 and Angpt2.

Lately, Rgp was reported as a pathogenic effector in the Alzheimer’s disease (AD). It degrades tau protein through proteolysis, which is a hallmark of AD (Dominy et al., 2019). This results in formation of insoluble and hyperphosphorylated tau, destabilization and dysfunction of microtubule structures, and ultimately synapse loss due to impaired axonal transport (Nixon et al., 2005; Miao et al., 2019; Dominy et al., 2019; Haditsch et al., 2020). It has also been reported that *P. gingivalis* releases Rgp-enriched outer membrane vesicles (OMVs) that are rapidly internalized into mammalian cells, causing NOD-, LRR-, and pyrin domain-containing protein 3 (NLRP3) inflammasome activation and the formation of apoptosis-associated speck-like protein containing a CARD (ASC) speck, which cause cell death *via* pyroptosis (Fleetwood et al., 2017). Of note, the activation of NLRP3 inflammasome in microglia and ASC specks initiates amyloid-β assembly and deposition (Venegas et al., 2017). Intraneuronal Rgp may also activate neuronal NACHT, LRR, FIIND, CARD domain, and PYD domains-containing protein 1 (NLRP1), resulting in neuronal cell death and caspase-1 activation, and the production of neuroinflammatory interleukins (IL-1 and IL-18) (Dominy et al., 2019).

Another study links *P. gingivalis* infection to hyperglycemia observed in diabetes mellitus cases (Seyama et al., 2020). *In vitro*, *P. gingivalis* has been shown to disrupt hepatic glycogen production by generating insulin desensitivity in liver cells and disabling the Akt/GSK-3 signaling pathway (Ishikawa et al., 2013). Seyama et al. (2020) corroborated the above finding by revealing that *P. gingivalis* outer membrane vesicles (OMV) are transported to the liver to impair insulin sensitivity. The OMVs affect hepatic glycogen synthesis by attenuating glycogen synthesis and insulin sensitivity in liver cells, resulting in an increase in blood glucose level (Seyama et al., 2020). Furthermore, P. gingivalis OMVs may also potentially modulate inflammation and immunological responses in macrophages by activation of the inflammasome complex and the generation of pro-inflammatory cytokines (Cecil et al., 2017), which could inhibit glycogen synthesis in hepatocytes as well. Additionally, Rgp causes significant alteration in pancreatic islet organization associated with β-cell apoptosis through upregulation of SerpinE1 (Ilievski et al., 2017). Apoptosis of pancreatic β -cells eventually leads to a decrease in functional β-cells that make insulin, resulting in Type 2 diabetes (Tomita, 2016).

Rgp is also linked to infectious disease (**Table 2**), such as aspiration pneumonia. It induces neutrophils infiltration into lungs by increasing IL-17 production, which may aggravate the local inflammatory response during lung infection, resulting in tissue damage, hemorrhage, and necrosis (Benedyk et al., 2015). Using the wild type W83 *P. gingivalis* strain alongside gingipain-deficient mutants, researchers suggested that Rgp activity in the lungs induces intrapulmonary bleeding *via* multiple activities on the respiratory epithelium and endothelial lining of capillary blood vessels. The study emphasizes neutrophil accumulation in lung tissue as a result of Rgp activity, which exacerbates inflammatory reactions in the lungs. (Benedyk et al., 2015).

**Table 2:** Rgp-related diseases and its mechanism of action.

Rgp-related diseases	Mechanism of actions	References
Periodontitis	* Activate kallikrein/kinin pathway; * Dysregulate clotting and fibrinolytic pathways; * Dysregulate host cytokine network; * Degrade complement components; * Activate MMPs	Imamura (2003); Fitzpatrick et al. (2009)
Cardiovascular disease	* Modify LDL by degrading apoE and apoB-100; * Increase oxidative stress and cause LDL peroxidation; * Modulate expression of Angpt1 and Angpt2	Zhang et al. (2015); Lönn et al. (2018)
Aspiration pneumonia	* Induce influx of neutrophils into lung tissue; * Activate platelets to release MCP-1, cause intrapulmonary hemorrhage and tissue necrosis	Benedyk et al. (2015)
Alzheimer’s disease	* Degrade tau protein; * Activate caspase-3 which act on tau; * Cause hyperphosphorylation of tau and synapse loss; * Release OMVs that drive NLRP3 inflammasome activation and ASC speck formation, which induce Aβ plaque formation; * Activate neuronal NLRP1	Dominy et al. (2019); Haditsch et al. (2020)
Diabetes mellitus	* Release OMVs with uptake by liver, which affects hepatic glycogen synthesis; * Inhibit Akt and GSK-3β signaling pathway; * Modulate inflammation and immune system in macrophages, * Alter organization of pancreatic islet, which leads to β-cell apoptosis	Ilievski et al. (2017); Seyama et al. (2020)
Preterm birth and low birth weight	* Degrade placenta and the fetus membrane; * Reduce IFN-γ production levels; * Increase TNF-α and IL-6 levels; * Alter helper T cell differentiation	Takii et al. (2018); ; Ao et al. (2015)

Besides, Rgp is also associated with pre-term birth and low birth weight. The protein could degrade placenta and the fetus membrane due to its strong collagenolytic activities (Abe et al., 1998). Takii et al. (2018) investigated this *in vivo* (in pregnant mice models) using two *P. gingivalis* strains, ATCC33277 and KDP136, the former being a wild-type strain and the latter being a Rgp-deficient mutant. Findings revealed that the ATCC33277 group had only one pre-term birth and no live pups as compared to the 90% survival rate of pups observed in KDP136 control group (Takii et al., 2018). Although ATCC33277 infection did not directly cause the mice’s preterm birth and low birth weight, but it did precede suppression of IFN-producing cells. A shift in the Th17/immune-suppressive Foxp3+ regulatory T cells (Treg) balance suppressed IFN-producing cells, resulting in elevated placental TNF and IL-6 levels and altered helper T cell development (Rowe et al., 2011; Figueiredo and Schumacher, 2016; Takii et al., 2018). Unfortunately, Takii et al. (2018) were unable to verify the link between *P. gingivalis* infection and suppression of IFN-γ levels. Despite that, the group hypothesized that *P. gingivalis* infections provided opportunities for subsequent infections by other bacteria or viruses that could directly contribute to pre-term births and low birth weight.

Altogether, Rgp could be considered as a prospective drug target to treat the aforesaid disorders. In fact, a growing number of investigations, ranging from *in vitro* to *in vivo* trials, have been reported on the suppression of Rgp by various types of inhibitors. These studies are discussed in more detail at the following sections.

### *In vitro* inhibition of Rgp using natural and synthetic compounds

To date, many gingipain inhibitors had developed to reduce the virulency of *P. gingivalis* (**Table 3**) so as to repress bacterial growth within the oral cavities, thus preventing the occurrence of PD. Majority of the inhibitors were from the natural sources, especially plant-derived compounds. In 1999, malabaricone C isolated from nutmeg was tested for its inhibitory activity against Rgp protease, recording an IC_50_ value of 0.7 µg/mL (Shinohara et al., 1999). Subsequently, many other plant extracts were also experimented for the same. Amongst them, polyphenolic plant bioactives (catechins and proanthocyanidins) were found to be effective inhibitors (Okamoto et al., 2004; Beckert and Hensel, 2013). Other plant extracts demonstrating gingipain protease inhibition activities include *Oryza sativa* (Taiyoji et al., 2013), *Dodonaea viscosa* (Patel et al., 2013), *Rumex acetosa* L. (Beckert and Hensel, 2013; Schmuch et al., 2015), *Canavalia gladiata* (Nakatsuka et al., 2014), *Rhododendron ferrugineum* (Löhr et al., 2015) and *Limonium brasiliense* (de Oliveira Caleare et al., 2017). Limitation in most of these studies is the lack of information pertaining to the inhibitory mechanism of Rgp. Plant extracts exhibiting inhibitory potential should also be analysed further (eg: bioactivity-guided fractionation) in order to determine the specific phytocompound(s) that contributed to the observed phenomenon.

**Table 3:** *In vitro* inhibition of Rgp by natural and synthetic compounds as well as other chemicals.

Category of Inhibitors	Examples	Effect	References
**Natural Products**	Malabaricone C (isolated from nutmeg *Myristica fragrans*)	Inhibition of protease activity	Shinohara et al., 1999
	Green tea catechins	Inhibition of protease activity	Okamoto et al., 2004
	Cranberry polyphenol fraction	Inhibition of protease activity	Yamanaka et al., 2007
	Polyphenols from *Myrothamnus flabellifolia*	Inhibition of protease activity	Löhr et al., 2011
		Upregulation of gene expression	
	Combination of A-type cranberry (AC-PACs) and licochalcone A	Collagenase activity inhibition	Feldman and Grenier, 2012
	Rice (*Oryza sativa*) protein extract	Inhibition of protease activity	Taiyoji et al., 2013
	Crude methanolic extract of *Dodonaea viscosa* var. angustifolia leaves	Inhibition of protease activity	Patel et al., 2013
	Proanthocyanidin-rich acetone-water extract (7:3) of *Rumex acetosa* L. (Polygonaceae)	Inhibition of protease activity	Beckert and Hensel, 2013
	Sword bean (*Canavalia gladiata*) extract	Inhibition of protease activity	Nakatsuka et al., 2014
	CL(K25A) dodecapeptide from rice protein	Inhibition of protease activity	Taniguchi et al., 2014
	Aqueous extract and polysaccharides from *Rhododendron ferrugineum* L.	Inhibition of protease activity	Löhr et al., 2015
		Downregulation of gene expression	
	Theaflavins	Inhibition of protease activity	Kong et al., 2015
		Collagenase activity inhibition	
	Proanthocyanidin-enriched extract from *Rumex acetosa* L. extract	Inhibition of protease activity	Schmuch et al., 2015
	Green tea extract and epigallocatechin-3-gallate (EGCG)	Downregulation of gene expression	Fournier-Larente et al., 2016
	Acetone: water extract (LBE) from the rhizomes of *Limonium brasiliense* (Boiss.)	Inhibition of protease activity	de Oliveira Caleare et al., 2017
	Eugenol from essential oil of *Syzygium aromaticum* (L.) Merr. & L. M. Perry (clove) leaf	Downregulation of gene expression	Zhang et al., 2017
	Quantum cucurmin	Inhibition of protease activity	Singh et al., 2018
	Epimedokoreanin B isolated from aerial parts of Epimedium species	Collagenase activity inhibition	Kariu et al., 2019
		Inhibition of protease activity	Kariu et al., 2016
	Thymoquinone	Downregulation of gene expression	Tantivitayakul et al., 2020
**Synthetic Compounds**	D-Phe-Pro-Arg-CH_2_Cl	Inhibition of protease activity	Potempa et al., 1997
	D-Phe-Phe-Arg-CH_2_Cl	Inhibition of protease activity	
	Benzamidine derivatives	Inhibition of protease activity [activity enhanced by Zn (II)]	Krauser et al., 2002
	KYT-1	Inhibition of protease activity	Kadowaki and Yamamoto, 2003
**Antiseptic**	Tetracycline and its analoques (doxycycline and minocycline)	Inhibition of protease activity	Imamura et al., 2001
			Grenier et al., 2003
	Chlorhexidine	Inhibition of protease activity [activity enhanced by Zn (II)]	Cronan et al., 2006
**Others**	Leupeptin	Collagenase activity inhibition	Houle et al., 2003
	Egg yolk antibody	Inhibition of protease activity	Yokoyama et al., 2007
	Pancreatic Kazal‐type trypsin inhibitors (pancreatic secretory trypsin inhibitors)	Inhibition of protease activity	Bania et al., 2008

Besides directly reducing the gingipain protease activity, the plant-derived compounds may modulate gene expression of gingipains in *P. gingivalis* as well. Polyphenols from *Myrothamnus flabellifolia* (Löhr et al., 2011) and green tea (Fournier-Larente et al., 2016) were experimented for such effects through microarray and quantitative PCR analysis, respectively. Microarray expression data showed a four-fold decrease in the mRNA expression of Rgp in *P. gingivalis* after treatment with the proanthocanydin-rich *Myrothamnus flabellifolia*. Similarly, the green tea extract, at a dosage of 75 mg/mL, demonstrated more than 50% reduction in gene expression of Rgp (Fournier-Larente et al., 2016). Recently, a plant derived monoterpene from black cumin, thymoquinone, was found to downregulate the gene expression of Rgp which eventually led to the inhibition of biofilm formation and hemolytic activity in *P. gingivalis*, thereby reducing its virulency (Tantivitayakul et al., 2020).

Apart from the natural products, small peptides like KYT-1 (Kadowaki et al., 2004) and chemical compounds such as benzamidine derivatives (Krauser et al., 2002) were also synthesized as gingipain inhibitors. Common antibiotics such as tetracycline, minocycline and doxycycline, were also shown to be good inhibitors of arginine specific gingipains (Imamura et al., 2001). Among the three antibiotics, the latter was proven to be a strong inhibitor with IC_50_ of 3 µM. Nevertheless, these group of inhibitors were shown to exhibit broad spectrum inhibition towards other proteases that may cause adverse effects to host cell, thus making the naturally occurring gingipain inhibitors, a better therapeutic agent (Olsen and Potempa, 2014). Besides plant extracts, other natural inhibitors of this protease were leupeptin, derived from actinomycetes (Houle et al., 2003) and an egg yolk antibody (Yokoyama et al., 2007).

### *In vivo* inhibition of Rgp using synthetic compounds

Besides using chemical-based or molecular-based techniques *in vitro* to determine Rgp inhibition, there are also experiments conducted *in vivo* specifically on murine model systems. However, only a small number of Rgp inhibitors are suitable for *in vivo* studies due to their non-reactivity with other essential host proteases (Olsen and Potempa, 2014). KYT1, a peptide analog, remains a popular candidate in several *in vivo* studies due to its low toxicity and high selectivity for Rgp.

Widziolek et al. (2016) employed Zebrafish larvae to showcase the link between Rgp and cardiovascular diseases *in vivo*. *P. gingivalis* travelled to different parts of the host *via* bloodstream and invaded vascular barriers, allowing their invasion into the surrounding tissue. The severity and mortality of cardiovascular illness were dose-dependent in *P. gingivalis*-infected zebrafish larvae, whereas gingipain knocked out *P. gingivalis* mutants-infected larvae showed substantially lower morbidity and mortality. More importantly, *P. gingivalis*-infected larvae, when treated with KYT-1, had significantly lower mortality and a higher survival rate than untreated groups, 48- and 72-hours post-infection.

Liu et al. (2017) explored the impact of Rgp on the activation of brain resident microglia. The study revealed that post-injection of *P. gingivalis* into the mice somatosensory cortex, microglia gathered at the injection site. However, when KYT-1 was injected alongside *P. gingivalis*, there was a considerable reduction in microglia accumulation around the injection site.

Dominy et al. (2019) recently proposed that *P. gingivalis* contributed to the etiology of Alzheimer’s disease (AD) by promoting neuronal damage *via* Rgp. COR286, a RgpB inhibitor, was administered orally to mice infected with *P. gingivalis* in this study. The results demonstrated that the bacterial burden in the brain was dramatically reduced, as was the loss of hippocampal Gad67+ interneurons. This finding suggested that inhibiting Rgp decreased *P. gingivalis* colonization in brain and subsequent neurodegeneration.

## *Porphyromonas gingivalis* peptidyl arginine deiminase

### Structural biology of PPAD

Full-length PPAD is organised into four domains, which include a N-terminal signal peptide, a catalytic domain, an immunoglobulin-superfamily domain (IgSF) and a C-terminal domain (McGraw et al., 1999; Goulas et al., 2015; Montgomery et al., 2016). It is found in the outer membrane fractions of *P. gingivalis*, and is observed to be truncated at the C-terminal and N-terminal domains (McGraw et al., 1999; Konig et al., 2014). According to the study by Konig et al. (2014), the truncated N-terminal found in PPAD is shown to benefit *P. gingivalis*, whereby the N-terminal serves to protect PPAD from autocitrullination. The ability of N-terminal truncation for PPAD preservation is further investigated by expressing full-length PPAD alongside N-terminal truncated PPAD in *Escherichia coli*, with results showing that full-length PPAD was autocitrullinated but truncated PPAD was not (Konig et al., 2014).

**Figure 4:**
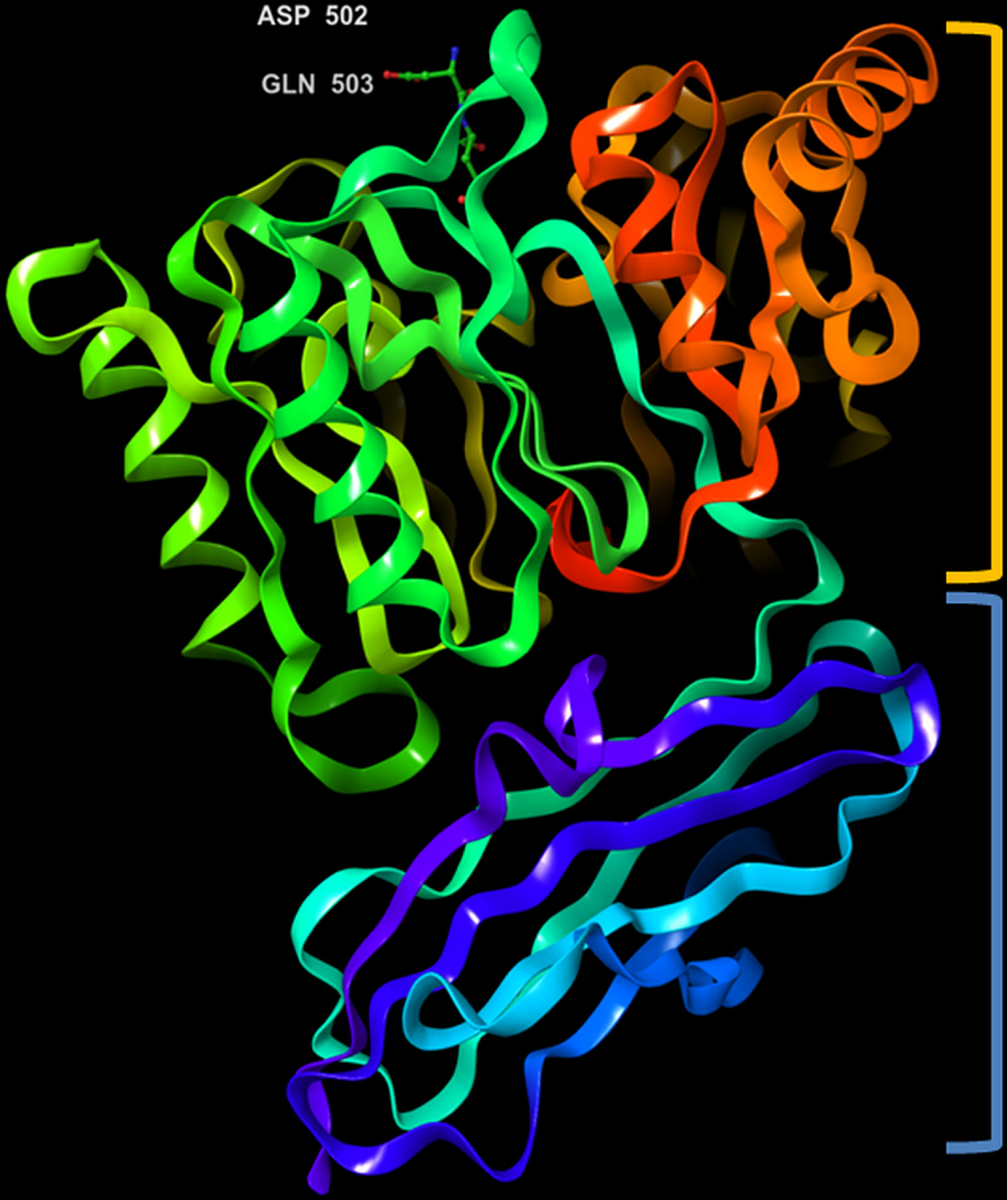
3D representation of *Porphyromonas gingivalis* PPAD with co-crystallized ligand, Asp-Gln dipeptide (substrate-mimic complex) (PDB ID: 4YTB). Highlighted in orange: crown (catalytic domain), blue: root (IgSF domain).

**Figure 5:**
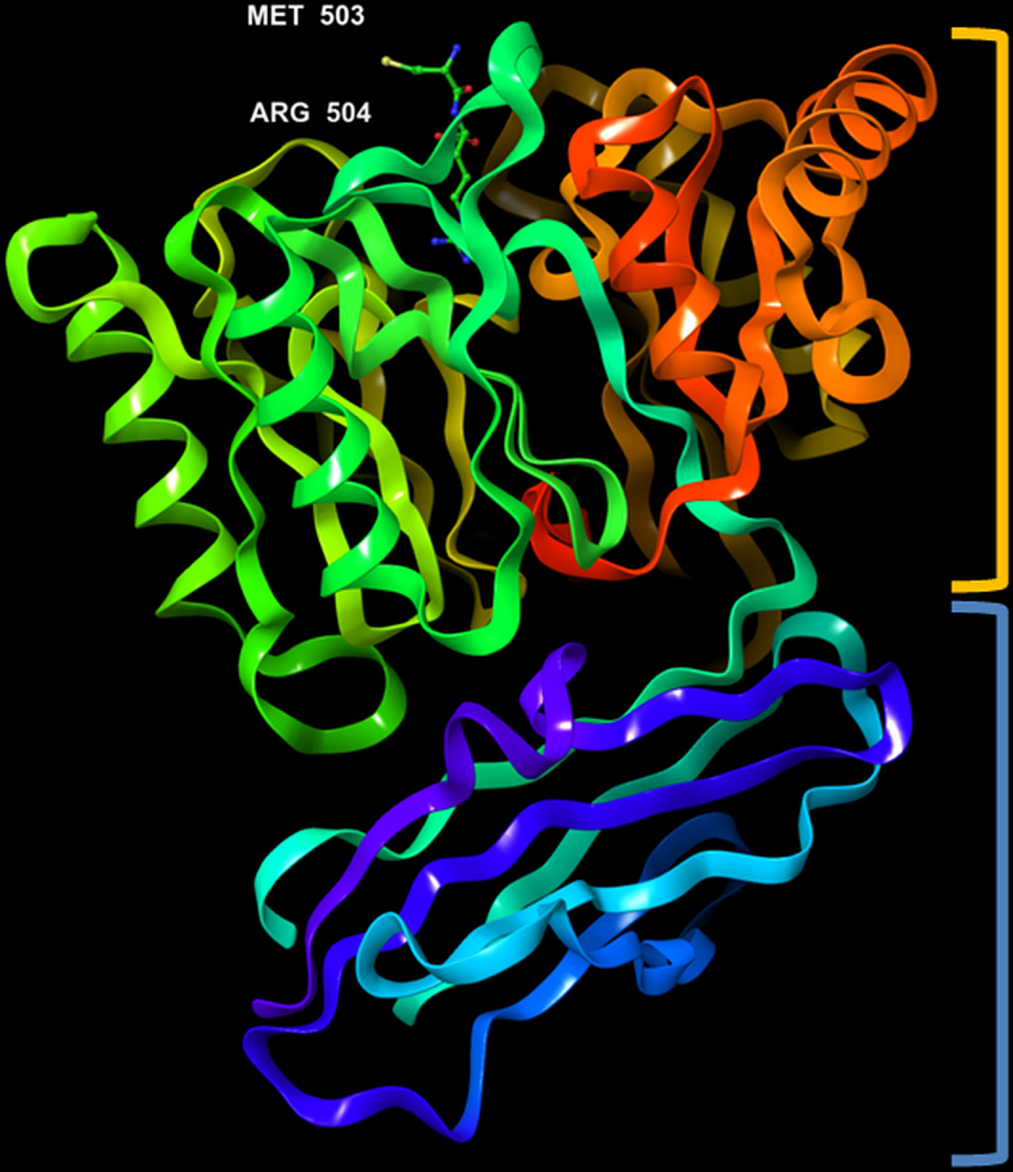
3D representation of *Porphyromonas gingivalis* PPAD with co-crystallized ligand, Met-Arg dipeptide (substrate complex) (PDB ID: 4YTG). Highlighted in orange: crown (catalytic domain), blue: root (IgSF domain).

The crystal structure of PPAD was found to be without any bound calcium ion, indicating that such ion is not needed for activity. Analogous to the Rgp, the structure resembles a tooth consisting of a crown and a root, which are linked by a connecting neck (**Figure 4** & **5**). The crown contains the catalytic domain (residues Ala44-Lys359) that includes an N-terminal extension and a α/β-propeller of five blades (I-V) arranged around a central shaft; each blade consists of a three-stranded twisted β sheet and one helix. The root is made up of a C-terminal immunoglobulin superfamily domain (residues Gly360-Glu465), which irregular β-sandwich with a mixed front sheet and an antiparallel back sheet (Eichinger et al., 1999; de Diego et al., 2013; de Diego et al., 2014). The topology and strand connectivity of such IgSF is similar to that of Rgp (Goulas et al., 2015; Montgomery et al., 2016), except for its length which is longer in PPAD than in Rgp (Goulas et al., 2015).

The active site cleft is situated in the centre of the α/β-propeller. It is a tapered, funnel-like hole, in which the arginine side chain of substrate is located. Such cleft is delineated by loops connecting the propeller blades and consists of highly conserved amino acids, such as Asp130, His236, Asp238, Asn297 and Cys351; the sulfur atom of Cys351 acts as catalytic nucleophile in the catalysis (Goulas et al., 2015; Montgomery et al., 2016). Gawron et al. (2017) compared the structure of a catalytically inactive C351A mutant of PPAD in complex with arginine substrate (PDB ID: 4YTG) to that of a wild type PPAD in complex with dipeptide aspartate-glutamine mimicking as the arginine substrate (PDB ID: 4YTB); consistently, it revealed that protein residues Asp130, His236, Asp238, Asn297, and Cys351 participated in ligand binding within the PPAD.

In addition, amino acids Arg152, Arg154 and Tyr233 are also found essential in interacting with the C-terminal main chain of substrate. Studies had shown that mutation of Arg152 and Arg154 led to the inhibition of enzyme activity, thus suggesting the role of both residues in determining the substrate specificity of PPAD for C-terminal argininyl peptide substrate (Montgomery et al., 2016; Rodríguez et al., 2010). Besides, it is inferred that substrate with C-terminal extension to the arginine was unable to bind in the binding pocket of PPAD as it will collide with Arg152 and Tyr233 side chains; this further corroborates the exodeiminase activity of PPAD against the C-terminal arginines. The substrate binding takes place once the surface loop (Michaelis loop’, residues Val226-Val237) is in an open conformation, which allows arginine residues of the substrates to gain access to the binding site. Once the substrate enters and binds in the active site, such loop changes the orientation and assumes a closed conformation (Goulas et al., 2015; Montgomery et al., 2016).

### PPAD-catalyzed citrullination as a unique feature of *P. gingivalis*

Human peptidylarginine deiminases (PADs) citrullinate proteins, such as trichohyalin, myelin basic protein, histones, and keratin/filaggrin present in hair follicles, central nervous system, nucleus, and skin, respectively (Briot et al., 2020). In addition, these PADs also play crucial roles in the regulation of vital processes of the human body, including gene expression, apoptosis, and inflammation (Olsen et.al., 2018).

There exist some differences between human PAD and PPAD. The bacterial protein has distinctive substrate specificity for C-terminal arginyl peptide, whereas human PADs can citrullinate peptides with internal arginines (Montgomery et al., 2016). Citrullination in general changes the spatial arrangement of 3D structure and function of proteins/peptides, thereby interfering with the host’s inflammatory signalling cascades (Olsen et.al., 2018). Human PADs regulate citrullination through calcium ion dependency and will cease citrullination activities when there is an absence of calcium ion. However, PPAD lacks a regulatory cofactor that can stop citrullination which potentially causes unregulated and unwanted citrullination of proteins in the host (Gawron et al., 2017; Olsen et al., 2018).

This PPAD-catalyzed citrullination has been linked, directly or indirectly, to various systemic disorders including PD, RA, AD, cardiovascular disorders, and atherosclerosis (Stobernack et al., 2016; Olsen et al., 2018). In particular, role of PPAD-catalyzed citrullination is most well studied in RA pathology. Byproducts of PPAD citrullination are classified and tagged as antigens by the host immune system, thereby triggering an immune response. As a result, the immune system produces anti-citrullinated peptide antibodies (ACPA), which are mostly found in the joints and contribute to RA clinical condition. Besides, ACPA expression can be found years before the onset of RA symptoms (Johansson et al., 2016). Notably, ACPA levels found in patients with RA show a proportional increase in tandem with progression of the disease, rendering such antibody as appropriate disease markers for RA (Johansson et al., 2016). In the case of PD, soluble PPAD enzyme modifies the epidermal growth factor, thereby interfering with its adequate interaction with epidermal growth factor receptor and leading to disruption of protective epithelial cell-periodontal tissue barrier and compromising the healing process (Pyrc et al., 2013). In addition, the presence of extensive citrullinated proteins, as observed in the myocardium of RA patients, atherosclerosis plaques, as well as in the hippocampal region of AD patients, also points towards the role of citrullinated proteins in cardiovascular disease and AD progression (Sokolove et al., 2013; Fert-Bober and Sokolove, 2014). Despite the extensive research done in this domain, only a little is known on how exactly PPAD-catalyzed protein citrullination is associated with these diseases.

### PPAD as causative agent of RA

Many murine model systems have been used to demonstrate that PD exacerbated the development of RA. *P. gingivalis* was found to connect periodontal disease to the severity of RA. Inoculation of other bacterial strain in the RA mice model such as *Escherichia coli* (Yamakawa et al., 2016) as well as other oral pathogens; *Prevotella intermedia* (Maresz et al., 2013) and *Fusobacterium nucleatum* (Jung et al., 2017), were unable to accelerate RA. From this viewpoint, we may extrapolate that the PPAD (found solely in *P. gingivalis*) is a crucial controlling factor. Other studies, focused on the immunity alteration effects of *P. gingivalis*, reported that this bacterium was found to downregulate B10 cells in arthritic DBA/1J mice, aggravating the disease condition (Zhou et al., 2021).

Nonetheless, effort was made to show citrullination by PPAD as a risk factor for RA in the *in vivo* systems. Yamakawa et al. (2016) reported that SKG mice with both PD and arthritis had three-fold higher levels of citrullinated proteins at diseased sites than those with only PD or arthritis at 6th week of study. According to the findings, higher citrullinated protein levels may enhance the course of RA by causing an increase in ACPA production. Another study found comparable results when Winstar rats were immunized with collagen Type II and then injected with *P. gingivalis*. Rheumatoid factor and citrullinated protein levels were reported to rise in rat serum and gingival tissue, respectively (Corrêa et al., 2017). Similar results were observed in DBA/1J arthritic mice induced with PD. When the researchers investigated further, fibronectin and enolase proteins which were both PPAD substrates, were found to be overexpressed in these mice as well. Using immunofluorescence labeling, these proteins were discovered to be co-localized with the citrullinated proteins (Jung et al., 2017). This study confirmed that *P. gingivalis* citrullination is a link between PD and RA.

Interestingly, the complete deletion of PPAD gene inhibited the citrullination process of *P. gingivalis* (Wegner et al., 2010). It was also recently shown that deleting PPAD hindered the synthesis of gingipain-containing outer membrane vesicles as well as their proteolytic activity (Vermilyea et al., 2021). As such, this enzyme could be regarded as a more potent target for inhibiting *P. gingivalis* compared to Rgp, specifically in the RA treatment.

## Conclusion and outlook

*Porphyromonas gingivalis* is involved in a multitude of systemic disorders, primarily due to its ability to citrullinate, which is aided by the presence of virulence factors, Rgp and PPAD. In the past, efforts made in this domain were directed towards inhibition of Rgp, a protease that cleaves off the protein substrates and subsequently exposes arginine residues for PPAD-mediated citrullination. However, recent discovery suggests PPAD as a more credible target for inhibition due to its ability to modulate the biogenesis of gingipain-containing outer membrane vesicles and regulate thier proteolytic activity (Vermilyea et al., 2021). In view of that, a PPAD inhibitor would be able to kill two birds with one stone, i.e., the inhibition of PPAD will also affect the activity of gingipain, Rgp.

Nevertheless, to date, only a single report exists on the inhibitory action of PPAD. In this work, leaf extract from *Cratoxylum cochinchinense*, constituting of mangiferin and vismiaquinone A, exhibited strong binding interaction with vital amino acid residues at the active site of PPAD *in silico* (Tan et al., 2021). More research is needed to fully explore the potential of PPAD as a therapeutic drug target. In this regard, virtual ligand screening and profiling strategies are indeed helpful. Explicitly, online drug databases can be filtered and screened for drugs that have been FDA approved before proceeding further to *in vitro* or *in vivo* testing. Databases to obtain FDA-approved drugs include, but are not limited to, DrugBank (Wishart et al., 2007), ChemBank (Seiler et al., 2007), PubChem (Kim et al., 2018), ZINC15 (Sterling and Irwin, 2015) and Therapeutic Target Database (Wang et al., 2020). FDA-approved drugs from these databases can be screened using *in silico* strategy for their therapeutic potential against *P. gingivalis* virulence proteins (Halperin et al., 2002). A typical drug discovery pipeline tends to be time consuming and costly. FDA approval process for newly developed drugs took an average of 12 years (Van Norman, 2016) and roughly 800 million to 1 billion U.S dollars were spent for every successful FDA approved drug (Khurana et al., 2018). As such, the drug repurposing approach, i.e., finding new uses for existing pharmaceuticals, could be of immense utility. This technique has demonstrated its effectiveness in lowering the cost and duration taken to complete the process of drug development. Recently, FDA-drugs had been repurposed as treatment for Alzheimer’s disease (AD) (Kumar et al., 2017) and also for COVID-19 (Chen et al., 2020; Ibrahim et al., 2020). Therefore, this strategy certainly would accelerate the discovery of potential inhibitors for this enzyme.

In addition to the *in silico* research, *in vitro* and *in vivo* validation of proposed therapeutics are critical. This is primarily owing to the intricacy of these systems, which ultimately can have a significant impact on medication response. In context of the present article, a drug should specifically target PPAD from *P. gingivalis*; off-targeting of drugs can have a negative impact on the normal physiological activity of neighbouring cells. In view of that, development of appropriate tissue engineered disease models, such as PD and other *P. gingivalis*-induced systemic disorders models, could pave the way for reliable pre-clinical analysis. For instance, a recent study developed a 3D vascularized human gingival model to assess the invasion of *P. gingivalis* into the blood streams (Sasaki et al., 2021).

Apart from that, a clear understanding of *P. gingivalis* mechanism of action and their pathophysiology might also facilitate the development of technologies for disease diagnostics and management. Despite the fact that PCR-based detection strategy is one of the simplest approaches for *P. gingivalis* diagnosis, it has certain drawbacks such as large sample requirement, time-consuming, labour-intensive and low sensitivity as compared to other technologies. In this regard, a recent trend is directed towards fabrication of point-of-care biosensing devices, varying from electrochemical, optical, colorimetric, and immunosensors. These sensors mostly exploit gingipains activities or cell surface markers for *P. gingivalis* detection (Nakka et al., 2015; Alhogail et al., 2018; Thonthat et al., 2019; Svärd et al., 2020; Park et al., 2021) but none of them is using PPAD. Hence, a huge scope exists for further development in this domain.

Given the severe consequences of *P. gingivalis* infection, there is a dire need for medical practitioners, pharmacologists, clinicians, and biomedical engineers to work together in order to thoroughly understand the interplay between the bacterial virulence factors especially PPAD with host proteins to cause a myriad of diseases.

## Author contributions

S-AT and SLC. contributed to conception of manuscript. YCC, S-AT, SLC, HCY, BG and YYO. wrote the first draft of this paper. YCC, S-AT, SLC, HCY, WYL, WYW, YYO. and TA reviewed and edited the manuscript. All authors read and approved the submitted version. 

## Funding

The authors wish to thank Tunku Abdul Rahman University College (TAR UC) and International Medical University (IMU) for financial support through TAR UC Internal Research Grant Scheme, project number: UC/I/G2020-00062, and IMU Internal Research Grant, project number: BP I-01-2022(10).

## Conflict of interest

The authors declare that the research was conducted in the absence of any commercial or financial relationships that could be construed as a potential conflict of interest.

## Publisher’s note

All claims expressed in this article are solely those of the authors and do not necessarily represent those of their affiliated organizations, or those of the publisher, the editors and the reviewers. Any product that may be evaluated in this article, or claim that may be made by its manufacturer, is not guaranteed or endorsed by the publisher.

## Abbreviations

Å, Angstrom; ACPA, Anti-citrullinated peptide antibody; AD, Alzheimer’s disease; ASC, Apoptosis-associated speck-like protein containing a CARD; BLASTP, Protein-Basic Local Alignment Search Tool; CARD, Caspase activation and recruitment domain; CCP, Cyclic citrullinated peptide; CIA, Collagen-induced arthritis; CII, Collagen type-2; EGCG, epigallocatechin-3-gallate; FDA, Food and Drug Administration; FFRCMK, D-Phe-Phe-Arg-chloromethylketone; FIIND, Function-to-find domain; HDL, High density lipoprotein; ICG, Indocyanine green; LDL, Low density lipoprotein; LRR, Leucine-rich repeat; MCP, Monocyte chemoattractant protein; MMP, Matrix metalloproteinases; NHI, National Health Insurance; NLRP1, NACHT, LRR, FIIND, CARD domain and PYD domains-containing protein 1, NLRP3, NACHT, LRR, FIIND, CARD domain and PYD domains-containing protein 3, NOD, Nucleotide-binding and oligomerization domain; OMV, Outer membrane vesicle; PAD, Peptidylarginine deiminase; PAC, Proanthocyanidins; PD, Periodontitis; PPAD, *Porphyromonas gingivalis* PAD; PYD, Pyrin domain; RA, Rheumatoid arthritis; Rgp, Gingipain R; AoSMC, Aortic smooth muscle cells; VLDL, Very low density lipoprotein.
